# Small Animal ^31^P Cardiac MR Spectroscopy: Sequence and Setup Optimization

**DOI:** 10.2174/0115734056420201251206075322

**Published:** 2026-03-06

**Authors:** Matthias Lütgens, Tobias Lindner, Cajetan Lang, Praveen Vasudevan, Brigitte Vollmar, Marc-André Weber, Stefan Polei

**Affiliations:** 1 Institute of Diagnostic and Interventional Radiology, Pediatric Radiology and Neuroradiology, Rostock University Medical Center, Rostock, Germany; 2 Core Facility Multimodal Small Animal Imaging, Rostock University Medical Center, Rostock, Germany; 3 Centre for Sports Cardiology/EAPC, University Hospital rechts der Isar, Munich, Germany; 4 Institute for Experimental Surgery, Rostock University Medical Center, Rostock, Germany

**Keywords:** MRS, Small animal, ^31^P, x-nuclei, Myocardium, Free induction decay

## Abstract

**Introduction:**

To determine the metabolic composition of mouse myocardium *in vivo*, the phosphorus (^31^P) magnetic resonance spectroscopy (MRS) setup was investigated and optimized.

**Materials and Methods:**

After assessment of coil properties, typical spectroscopy sequences were evaluated against each other using metabolite phantoms of adenosine triphosphate (ATP) and phosphocreatine (PCr) on a Bruker 7 T small animal scanner, with respect to signal-to-noise ratio (SNR) and spatial selectivity.

**Results:**

The free induction decay (FID) in combination with outer volume suppression (OVS) showed the best SNR compared to the other spectroscopy sequences. OVS achieved a signal suppression of adjacent metabolites of approximately 84%. The performance of the setup was demonstrated in beating mouse hearts before and after myocardial infarction. A PCr to ATP(γ) ratio change from 2.5 pre-infarction to 1.74 approximately one week post-infarction was observed.

**Discussion:**

Among the evaluated methods, FID with OVS provided the best balance between localization accuracy and acquisition efficiency for application in the beating mouse heart. Despite probable signal contamination from surrounding tissue, *in vivo* measurements revealed post-infarction metabolic changes, evidenced by the reduction in the PCr/ATP(γ) ratio.

**Conclusion:**

Due to the dynamic nature and small size of the target organ, examination of the beating heart with ^31^P MRS requires a signal intensity- and noise-optimized implementation. The FID with OVS approach allows clear detection of variations in the ATP to PCr ratio before and after myocardial infarction within an acceptable measurement time (approximately 6.5 min). Image-selected *in vivo* spectroscopy (ISIS), the only serious competitor to FID with OVS, was found to be less suited for *in vivo* myocardium investigations due to inherent limitations in signal intensity and SNR.

## 1 INTRODUCTION

The entire cell metabolism is closely linked to the chemical energy supply *via* metabolites. Key players in this context include adenosine triphosphate (ATP), which exhibits three distinct resonances in the ^31^P nuclear magnetic resonance (NMR) spectrum — γ-ATP, α-ATP, and β-ATP — as well as phosphocreatine (PCr). Knowledge of the interplay between these metabolites provides deep insight into the cellular energy budget, as an imbalance between the supply and demand of ATP can contribute to disease [1]. For this reason, there is considerable interest in non-invasive *in vivo* quantification of metabolite concentrations or metabolite ratios.

A possible approach to achieve this insight is ^31^P magnetic resonance spectroscopy (MRS) [2], where the signal is generated by phosphorus nuclei rather than hydrogen nuclei. However, because ^31^P metabolites have much lower tissue concentrations and a smaller gyromagnetic ratio compared to ^1^H, the resulting MR signal intensity from ^31^P nuclei is typically about four orders of magnitude lower than that of ^1^H [3].

Recent advances in small-animal ^31^P MRS have focused on improving spatial resolution, signal-to-noise ratio (SNR), and metabolic quantification in the murine heart. Maguire *et al*. [4] demonstrated the feasibility of high-resolution *in vivo* cardiac ^31^P chemical shift MRS (CMRS) using a threefold under-sampled density-weighted chemical shift imaging (CSI) approach, enabling the detection of altered PCr/ATP ratios in transgenic mouse models despite prolonged acquisition times. Shaul *et al*. [5] combined ^31^P NMR spectroscopy with hyperpolarized ^13^C approaches in perfused mouse hearts to simultaneously monitor energetic status and pH, providing a comprehensive assessment of myocardial metabolism under ischemic conditions. These developments emphasize the ongoing need to optimize hardware, sequences, and scan parameters to achieve usable SNR in ^31^P MR measurements in small animals [6]).

Beyond methodological considerations, ^31^P MRS provides a direct functional window into cardiac pathophysiology. For instance, mitophagy has been shown to play a dual role in myocardial ischemia/reperfusion injury, being either protective or detrimental depending on the context [7]; alterations in PCr/ATP ratios could serve as sensitive *in vivo* correlates of these processes. Similarly, fibroblast activation is a recognized driver of cardiac fibrosis [8], and ^31^P MRS may allow non-invasive monitoring of the energetic consequences of pharmacological or genetic interventions targeting such remodeling pathways.

Given the growing potential of ^31^P measurements to non-invasively assess *in vivo* cardiac metabolism relevant to heart failure, ischemia-reperfusion injury, and cardiomyopathies, this study sought to re-evaluate the optimal combination of the key technical parameters influencing data quality and reproducibility in murine models.

## 2 MATERIALS AND METHODS

Data were acquired on a 7 T Bruker BioSpec 70/30 system (Bruker, Ettlingen, Germany). The setup was equipped with the B-GA12S gradient inset (max. amplitude: 440 mT/m, max. slew rate: 3440 T/m/s) and a double resonant ^1^H/^31^P transmit-receive surface coil with an inner diameter of 20 mm (T6615V3, Bruker, Ettlingen, Germany). For ^31^P sensitivity comparison, standard free induction decay (FID), stimulated echo acquisition mode (STEAM), point-resolved spectroscopy (PRESS), and image-selected *in vivo* spectroscopy (ISIS) sequences were used, with the parameters listed in Table **1**. Voxel sizes in the localized spectroscopy sequences were chosen so that the entire sample volume was included.

The 90° and 180° pulses were set to be as intense as possible to minimize pulse length without exceeding the maximum power limit of the surface coil, resulting in the pulse lengths shown in Table **1**. The default spoilers specified in the STEAM, PRESS, and ISIS sequences were applied. For the PRESS sequence, a minimum echo time (TE) of 14.92 ms was achieved. The total echo time was divided symmetrically between the two echo times, TE1 and TE2. Significantly shorter echo times were possible for the STEAM sequence; to investigate the effect of shorter TE on signal strength, STEAM sequences with three TE values were used: one identical to PRESS, a minimized echo time of TE = 2.62 ms, and an intermediate echo time of TE = 7.46 ms. A total of 64 averages was used for FID, STEAM, and PRESS. As the ISIS sequence requires eight individual measurements per run by design, only eight averages were applied here. This resulted in an identical measurement time of 192 s for all sequences.

Since FID spectroscopy is not spatially selective, six saturation slices were defined around a cube-shaped volume of interest (VOI) to obtain a spectrum primarily originating from the enclosed area, following the approach of Deschodt-Arsac *et al*. [9]. The size of the enclosed area in our approach was approximately 5 × 8 × 11 mm.

To evaluate the coil characteristics, a flexible ^1^H phantom (a water-filled laboratory glove) was used, which adapts well to the coil. Additionally, phantoms of ATP (200 mM) and phosphocreatine (PCr, 200 mM) in aqueous solution were prepared by dissolving ATP disodium salt (Sigma-Aldrich) and creatine phosphate disodium salt tetrahydrate (Carl Roth). Shimming was performed iteratively to minimize the linewidth of the ^1^H signal within the volume of interest (VOI), resulting in typical linewidths of approximately 10 Hz for the PCr peak in ^31^P phantom measurements. This local iterative shimming approach was also applied even when non-localized sequences were used to acquire the final spectra.

For *in vivo* measurements ^31^P FID MRS parameters were as follows: block pulse excitation with 0.08 ms duration and 90° flip angle, repetition time (TR) of 1200 ms, 350 averages, excitation and receiver bandwidth of 16 kHz, and six spoiling slices positioned around the heart (Fig. **4b**). FID and Fourier-transformed spectra contained ~2400 data points, and no zero-filling or baseline correction was applied.

All animal experiments were approved by the local animal care committee of the “Landesamt für Landwirtschaft, Leben-smittelsicherheit und Fischerei Mecklenburg-Vorpommern” (LALLF, Germany; Ref.Nr. 7221.3-1-018/21). Permanent myocardial infarction was induced through ligation of the left anterior descending coronary artery (LAD). MR scans were performed on one 10-week-old C57BL/6JRj female mouse (weight: 21g, Janvier Labs) five days before and approx. one week after infarct induction.

For the MR investigation, animals were anesthetized with a mixture of isoflurane and oxygen. During measurements, the isoflurane proportion was adjusted to maintain a respiration rate near 50 breaths per minute. Respiratory gating and prospective cardiac ECG triggering (ERT Gating Control Model 1030, SA Instruments Inc., Stony Brook, USA) were applied for all *in vivo* measurements. Body temperature was maintained at 37°C by positioning the mouse on a warm water–flushed silicon pad.

Prior to use, mice were housed in the animal facility of Rostock University Medical Centre under specified pathogen-free conditions, with food and water provided ad libitum. Following surgical procedures, mice were allowed to recover in individual cages. Environmental enrichment included nesting material (shredded tissue paper, Verbandmittel GmbH, Frankenberg, Germany), paper rolls (75 × 38 mm, H 0528–151, ssniff-Spezialdiäten GmbH), and wooden sticks (40 × 16 × 10 mm, Abedd, Vienna, Austria).

To minimize animal suffering, mice were examined daily using a scoring sheet. In cases of unrelievable pain, as determined by the score sheet, the animal was immediately euthanized by cervical dislocation.

## 3 RESULTS

### 3.1 Coil Characteristics

The characteristics of the used coil in ^1^H mode were investigated using signal-intensity data. Due to the much lower signal intensity, corresponding ^31^P images are not readily available. However, the statements presented here on the coil properties can also be applied to measurements in ^31^P mode, as the spatial characteristics of the coil with respect to B1 field distribution and receive sensitivity are assumed to be very similar for both the ^1^H and ^31^P channels.

To determine the area of homogeneous excitation of the surface coil in the half-space above the coil, the signal intensity distribution of a flexible water phantom was measured using a gradient echo (GE) sequence. A GE sequence was chosen because it employs only a single radiofrequency (RF) pulse, analogous to a simple FID measurement. To characterize the B1 field, parameters of a proton-weighted sequence were used: TR = 6 s, TE = 4.5 ms, and flip angle = 30°. Since the phantom is homogeneous, any observed signal variations can be attributed to inhomogeneities in the B1 field.

**Fig. (1):**
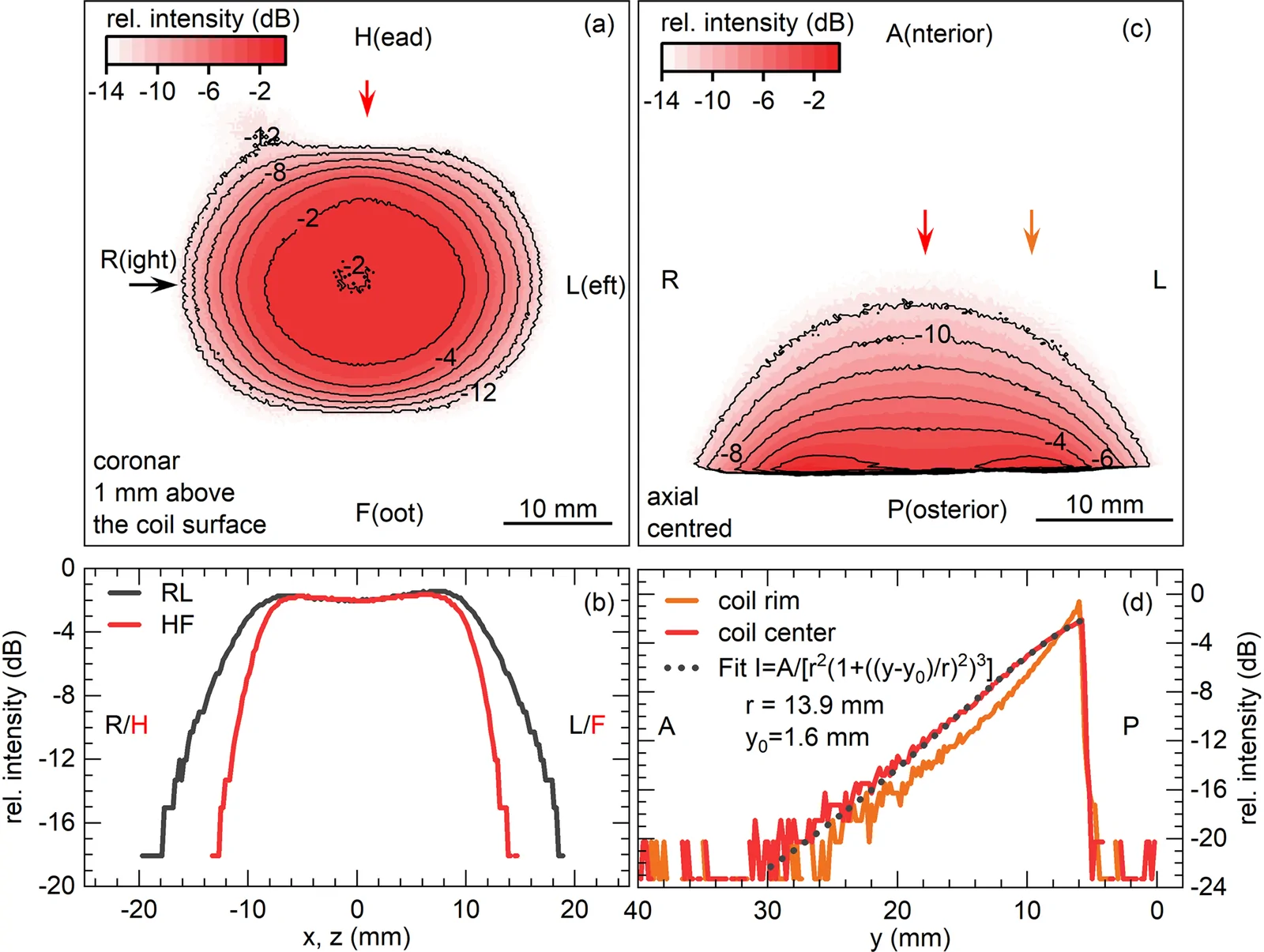
Proton signal of a homogeneous sample: (**a**) coronal section 1 mm above the coil; (**c**) axial sections approximately centered on the coil. (**b**) and (**d**) show intensity profiles, with positions indicated by the arrows in the respective sections. In addition, the signal drop in the direction perpendicular to the coil surface has been fitted with a model.

In Fig. (**1a**), the measured intensity distribution of a coronal slice parallel to the coil surface at a distance of 1 mm is shown. Isointensity lines reveal a rapid signal intensity drop near the rim of the coil and a roughly elliptical region of homogeneous signal intensity near the coil center. Line profiles along the left–right (x) and head–foot (z) directions, as shown in Fig. (**1b**), confirm an intensity plateau spanning approximately 17 mm in the z-direction and about 19 mm in the x-direction, with intensity variations less than 0.6 dB (15%). In the direction perpendicular to the surface, the B1 field decays as expected for a ring coil (see Fig. **1d**) [10, 11], effectively reducing sensitivity by a factor of 2 for every 5 mm in this direction. The decrease in signal intensity with increasing distance from the coil surface was further analyzed in axial slices Fig. (**1c**). Fig. (**1d**) shows line profiles from Fig. (**1c**) along the directions indicated by the red and orange arrows.

Note that the surface coil was used for both sample excitation and signal reception. Thus, the data shown in Fig. (**1**) reflect the combined effects of B1 field inhomogeneities during excitation and spatial variations in receive sensitivity, particularly in the direction perpendicular to the coil and in regions outside the active coil area. The magnetic field B1 along the central axis of a ring coil with radius r can be described by [11]:


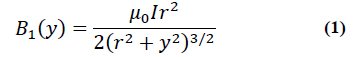


where *µ*_0_ is the permeability of free space, *I* the current, and *y* the axial distance from the center of the coil. Due to the reciprocity principle [12], the receive-sensitivity profile is proportional to the transmit field. Therefore, in the small-flip-angle regime, the detected signal scales approximately with the square of the B1 field, which leads to an axial signal decay proportional to 1/*y*^6^. Using Eq. **1** as the basis for signal analysis, the decrease shown in Fig. (**1d**) closely follows the characteristic fall-off of a ring surface coil.

Based on the profiles in Fig. (**1b** and **d**), it can be stated that the described plateau is only weakly developed. Signals from protons at a distance of 5 mm are already 50–60% smaller compared to layers directly at the coil surface (data not shown).

### 3.2 Sequence Sensitivity & SNR Comparison

In Fig. (**2**), the sensitivity—assessed by comparing the noise of maximum-intensity normalized data and the SNR—is presented. Using the normalized data, the noise was calculated as the standard deviation within a spectral range where the influence of the resonance is negligible (-10 ppm to -16 ppm).

For the SNR, the area under the resonance peak was determined in the range ∆*v* = *v*_0_ ± 1 ppm and then divided by the linewidth. This quotient of area and resonance linewidth represents the average signal strength. The noise was calculated as the standard deviation from the original spectra in the range from -10 ppm to -16 ppm. From the mean signal strength and noise, the SNR was derived.

The lowest noise and highest SNR are obtained for the signal from the FID. This is followed by ISIS, PRESS, and STEAM, with STEAM showing the highest noise and lowest SNR. The differences in noise (4%) and SNR (14%) between the FID and the ISIS measurements are relatively small. However, compared to the FID, the SNR for the PRESS measurement is reduced by 28%, while the STEAM measurements are on average 55% lower than the FID measurements. The noise for the PRESS measurements is 31% higher than that of the FID measurements, whereas the average noise for the STEAM measurements is 114% higher. Given that the sensitivity achieved with PRESS and STEAM was significantly lower than with FID or ISIS, both techniques are excluded from further investigations.

### 3.3 Sequence Selectivity Comparison

The selectivity tests aim to evaluate the ability of the methods to differentiate between spatially distinct regions that may each contribute to the measured signal. Two separate phantoms as shown in Fig. (**3a**) are used, each exhibiting unique spectral signatures that allow unambiguous attribution of signal contributions to their respective sources.

The spectra are shown in Fig. (**3b**). The black spectrum represents the ^31^P resonance of the PCr phantom, while the red bands correspond to the second phantom, consisting of an aqueous ATP solution. The ATP spectrum exhibits three well-resolved bands, each arising from one of its phosphate groups.

Selectivity is assessed by the extent to which one of the two signals, PCr or ATP, can be suppressed. As the signals originate from spatially separated samples, selectivity measures the ability to isolate signals from a defined measurement volume without contamination from surrounding regions. In ISIS, localization is achieved through a combination of individual measurements, where a set of selective 180° pre-pulses is applied in each measurement before the signal-generating 90° RF pulse. The addition of these individual signals results in the cancellation of signals from outside the region of interest (sequence pattern in [10]).

To enhance the spatial selectivity of the FID technique beyond the level specified by the coil characteristics, areas that should not be detected can be suppressed using 90° saturation slices. Here, multiple saturation pre-pulses are used in a single measurement to isolate the measurement volume *via* outer volume suppression (OVS). One 90° saturation pulse with a duration of 4 ms was applied as part of the standard FID protocol, referred to as “Single pulse” in the ParaVision software.

The phantoms used here consist of 0.5 ml microreaction vessels, positioned as close together as possible in the center of the coil. During the FID measurement, the ATP phantom could be completely covered with a sagittal 9 mm saturation layer. For the ISIS sequence, the measurement voxel was positioned entirely within the PCr sample. FID with OVS and localized ISIS are compared with spectra acquired without any localization technique.

The resulting spectra are shown in Fig. (**3c**). The two top spectra show the FID spectrum without OVS and the ISIS spectrum with a measurement voxel containing both phantoms. The line splitting in the ATP phosphate bands due to spin-spin coupling cannot be observed, as the shim volume was positioned in the PCr phantom, resulting in broadened signatures from signals originating from the ATP phantom.

The two lower spectra in Fig. (**3c**) show the results after using localized sequences. A saturation pulse was applied in the FID measurement to suppress the ATP component. For the ISIS sequence, the measurement voxel was reduced in size so that only the PCr sample was within the voxel. As a result, no ATP signal components are visible in the ISIS spectrum. In the FID spectrum, a reduction of the ATP contributions is observed; however, residual ATP signals above the noise level remain clearly perceptible. Quantitatively, the SNR of the PCr peak in the ISIS measurements (upper spectrum: SNR 95, linewidth 0.083 ppm; lower spectrum: SNR 127, linewidth 0.082 ppm) was on average 18% lower compared to the PCr signal in the FID spectra (upper spectrum: SNR 112, linewidth 0.089 ppm; lower spectrum: SNR 127, linewidth 0.099 ppm). This reduction in SNR is consistent with the initial measurements performed on single phantoms.

To quantify the residual signals, a slightly different analysis was performed because the linewidth of the ATP signal was difficult to determine. The spectra were normalized to the PCr resonance of the FID measurement without OVS. The reduction of the parasitic ATP signal was then determined using the ratio of the sum of the areas under the three ATP bands. Signal suppression with OVS was approximately 84%.

### 3.4 *In vivo* ^31^P MRS of Mouse Myocardium

Based on the results of the previous chapters, FID with OVS is expected to provide the best ^31^P results for *in vivo* mouse myocardium investigations (see Discussion for details). After fast multislice, multiplane anatomical reference scans in three orthogonal planes covering the heart and adjacent tissue, six saturation slices used for OVS were positioned around the heart, as shown in Fig. (**4b**).

Corresponding ^31^P MR spectra are shown in Fig. (**4a**). Spectra were smoothed by multiplication of the time-dependent signal with an exponential decay function, resulting in 33 Hz line broadening after Fourier transformation. The red spectrum represents a healthy mouse heart, while the black spectrum depicts the same mouse approximately 1 week after surgical myocardial infarction (LAD ligature).

A clear reduction in the PCr peak height of ∆~40% can be seen. Apart from a slight reduction of the PME/Pi/PDE region, the rest of the spectrum shows no remarkable changes. The full width at half maximum (FWHM) of the PCr peak in the healthy mouse is approximately 1 ppm, and the SNR of the spectrum is around 15 (evaluated with the TopSpin “SINO” tool).

To estimate the OVS efficiency, a seventh saturation slice was added above the heart, covering the area enclosed by the other saturation slices. A corresponding spectrum (same mouse, approximately 1 week post-operation) is shown in dashed gray in Fig. (**4a**). Comparison of the black and dashed gray spectra shows a PCr peak height difference of about 35%.

Notably, all FID spectra had an underground signal in the form of a broad Gaussian^1^ peak subtracted. This underground signal is attributed to low-mobility phospholipids originating from the residual bone/sternum signal superimposed on the heart signal, similar to findings in ^31^P MRS of human and rodent brains [13, 14]. Another possible explanation for this broad underground could be phase errors in combination with an unaccounted-for acquisition delay (50 µs after the end of the excitation pulse). However, by cutting off the first ~4 ms (acquisition delay & digitizer-specific delay, called “group-delay”) of the raw time-domain data until the onset of the actual FID and by thoroughly correcting the phase of the Fourier-transformed data, these confounding factors could be ruled out. An uncorrected spectrum (purple/red) including a fit (gray) of the underground signal is shown in the inset of Fig. (**4c**). For the fit, data points belonging to the underground were manually masked (purple).

**Fig. (4):**
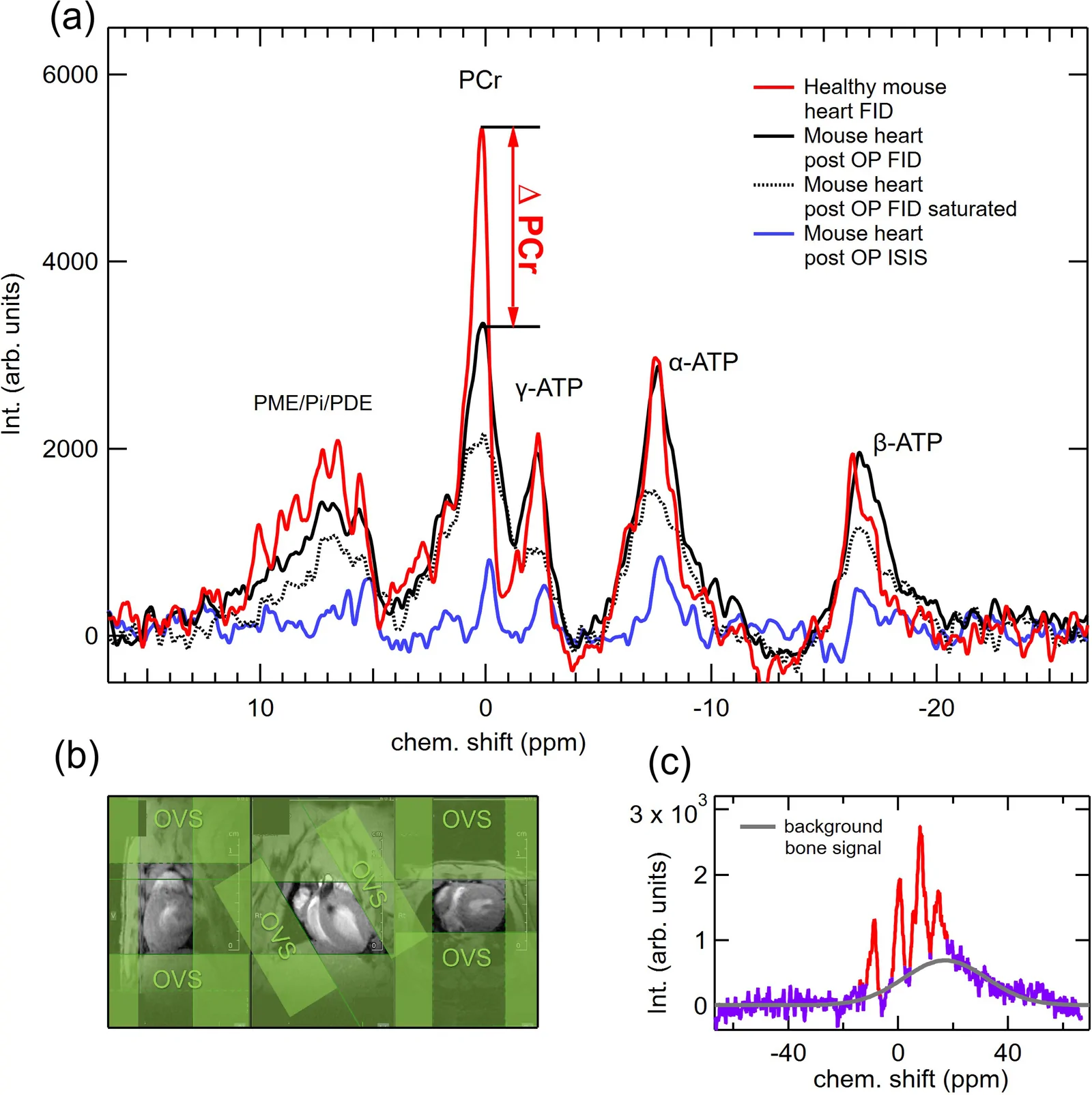
(**a**): ^31^P MRS of a 10-week-old female C57BL/6JRj mouse (21 g). Spectra of pre-infarction (red) and approximately one week post-myocardial infarction (all other colors) are shown. The resulting spectrum after applying an additional saturation slice over the heart is shown as dotted black. Blue spectrum: ISIS measurement of the same VOI. (**b**) Three-plane T1-weighted anatomy of the mouse heart with OVS visualization in green. (**c**) Determination of bone-phosphorus-lipid–related underlying signal.

For comparison, and because the OVS showed poor suppression performance at first glance, ISIS was also performed (blue spectrum in Fig. **4a**). However, at the same measurement time, the signal intensity and SNR of the ISIS were very poor and not considered suitable for *in vivo* MRS. Peak amplitudes of all ATP and the PCr peak were about a factor of ~ 4 smaller for the ISIS measurement when compared to the FID with the OVS approach, as shown in Fig. (**4**). Although in line with our previous assessment after the phantom investigations, the signal intensity difference between FID and ISIS was much more significant than in the phantom investigations.

It should be noted that a Gaussian profile was assumed for the peak shape here. However, Lorentz or Voigt profiles would also be acceptable, as the peak shape cannot be clearly defined due to noise.

## 4 DISCUSSION

### 4.1 Sequence Sensitivity & SNR Comparison

The investigations revealed clear differences between FID, ISIS, and PRESS measurements compared to the STEAM sequence. These differences arise because, in the STEAM sequence, a spoiler gradient applied after the initial 90° excitation pulse distributes the magnetization isotropically in the xy-plane. With the subsequent 90° pulse, only the y-component of this isotropic distribution of magnetic moments is rotated along the z-axis, resulting in a signal loss of 50% [10]. This signal loss is clearly reflected in the results presented: compared to the FID measurement, the noise of the STEAM measurement is more than doubled, and the SNR is more than halved. Compared to the PRESS sequence, the STEAM sequence offers the advantage of enabling very short TEs, which allows partial compensation of the signal loss. This effect was investigated by varying the echo time in the STEAM sequence. It was found that a reduction in TE from the original 14.92 ms to 2.62 ms was accompanied by a 14% reduction in noise and a 7% increase in SNR. Although this effect on noise and SNR was measurable, the sequence-intrinsic signal loss described above could not be fully compensated.

Compared to the STEAM sequence, the differences between FID, ISIS, and PRESS are significantly smaller. These differences are mainly due to the dephasing of magnetization. In the FID and ISIS sequences, the signal is measured directly after a 90° excitation, whereas an echo signal is recorded with PRESS. Since the first 90° excitation is followed by two more pulses before the echo is generated, the amplitude of the echo signal is weighted by the echo time TE *via* the factor exp (-TE/T2).

### 4.2 Sequence Selectivity Comparison

Although the ISIS spectrum shows the least contamination from areas outside the voxel compared to FID, it suffers from substantial signal loss if the voxel is positioned further away from the coil surface (data not shown). This is because deviations from the 180° flip angle required for maximum signal intensity [15] are more likely to occur at larger distances from the coil surface. Additionally, for application on the beating mouse heart, the many individual measurements required for the ISIS sequence are problematic. Either the data collection of the entire MRS experiment is prolonged because one has to wait for the trigger of the next cardiac cycle, or, if the eight individual sequences follow one after another, the heart motion during data acquisition results in the individual spectra being recorded at different periods of the cardiac cycle. Hence, FID with saturation slices is considered the best choice for application on beating mouse hearts.

Comparing our FID data with saturation slices to previous work [9] shows that our suppression was somewhat less effective: PCr could be reduced by a factor of approximately 10 in Deschodt-Arsac *et al*., but only by about a factor of 6 (~83%) in our experiment. This difference is most likely related to the lower linewidths achieved by Deschodt-Arsac *et al*. (<< 1 ppm) compared to ~1 ppm in our *in vivo* experiment. We attribute this to the lower field strength of our MRI system and the phantom vials used, which had diameters at least twice as large as those in [9], resulting in greater B0 disturbances, less effective shimming, and consequently broader linewidths. Additionally, the larger inner diameter of our surface coil may have caused greater B1 field inhomogeneity, resulting in more significant longitudinal residual magnetization after saturation, which can contribute to the measured signal [16].

### 4.3 *In vivo* ^31^P MRS of Mouse Myocardium

Comparing the peak height changes of spectra acquired with and without an additional saturation slab over the heart (gray and dotted gray in Fig. (**4a**) revealed an average (PCr and α−, β−, γ− ATP) reduction of approximately 50%. This is lower than the 84% reduction obtained in the phantom experiment, but this discrepancy is expected since the *in vivo* VOI contains the dynamic heart and several susceptibility interfaces, rather than a simple static phantom. Nevertheless, it emphasizes that the suppression efficiency of the saturation slice approach is limited and that contamination from metabolite signals outside the VOI is significant. Therefore, the measured metabolite peak ratios within a spectrum likely underestimate the true metabolite ratios in the heart.

Nevertheless, a direct comparison of pre- and post-myocardial infarction spectra revealed a significant reduction in the PCr peak height, clearly reflecting the metabolic stress following infarction. The number of myocardial cells was reduced, and the remaining cells experienced an increased workload to maintain pumping function, leading to an energy deficit [17-19]. This shift toward a lower energy reserve is consistent with a previous study [20] and underscores the potential of ^31^P MRS for monitoring heart failure progression and therapeutic interventions. While Maguire *et al*. [4] successfully applied CSI to obtain highly localized data, their approach required lengthy acquisition times of approximately 70 minutes, limiting its *in vivo* applicability in mice. In contrast, our FID + OVS implementation offers a time-efficient alternative (total measurement time ~30 min) with sufficient sensitivity to detect infarction-related metabolic changes, albeit with moderate contamination from surrounding tissues. Shaul *et al*. [5] further demonstrated the importance of combining ^31^P spectroscopy with complementary techniques, such as hyperpolarized ^13^C spectroscopy, to gain a more comprehensive picture of cardiac energetics and pH regulation. Together, these studies highlight the relevance of our approach as a practical compromise between acquisition speed and localization accuracy for routine small-animal cardiac studies.

For quantification, the PCr to ATP(γ) peak height ratio was determined to be 2.5 pre-infarction and 1.74 post-infarction. Thus, despite the limited efficacy of our saturation approach, the observed PCr / ATP(γ) peak height ratio changes are sufficient to track the metabolic response of the myocardium after infarction.

## 5 LIMITATIONS

The limitations of the phantom measurements arise from the static nature of the phantom compared to the motion of the heart, as well as from the unrealistic conditions introduced by the pronounced susceptibility difference between the phantom and the surrounding air, in contrast to the mouse heart, which is embedded in biological tissue.

The *in vivo* data include significant signal contamination from surrounding tissue and skeletal muscle. A more efficient suppression approach or sophisticated hardware, such as a dedicated crusher coil [21] or a smaller surface coil, might considerably improve signal quality. In this manuscript, the results of our approach to implement ^31^P spectroscopy in the mouse heart are demonstrated using exemplary measurements in a single mouse. For future investigations, a larger cohort will be required to obtain meaningful statistics on cardiac development after infarction.

The methodology presented here has been specifically optimized for the small mouse heart. In larger animals or clinical MRI systems, coil geometry, magnetic field homogeneity, and anatomical constraints differ substantially. In such cases, localized spectroscopy techniques targeting larger voxels within defined cardiac regions can be implemented. Although the FID with OVS approach is conceptually transferable to these settings, achieving adequate signal-to-noise ratio and spatial specificity would likely require advanced hardware and methodological adaptations, such as higher-order shimming, the use of dedicated crusher coils, or phased-array detection.

## CONCLUSION

In summary, we presented our approach to establish *in vivo* ^31^P MRS on mouse hearts. For this purpose, we investigated the characteristics of the dual-tuned ^31^P/^1^H Bruker surface coil with respect to excitation and receive sensitivity. The typical B1 decay of a ring coil and a weakly developed plateau of constant signal intensity were observed. Subsequently, different spectroscopy approaches (STEAM, PRESS, ISIS, FID) were compared. With respect to SNR, STEAM and PRESS were found unsuitable for the planned application. The following selectivity investigation demonstrated superior performance of ISIS compared to FID with OVS. Nonetheless, ISIS was ruled out due to the more pronounced signal loss at larger distances from the coil surface and the error-prone requirement of synchronizing the eight individual ISIS partial measurements with the cardiac cycle. Finally, we showed that, despite the somewhat limited effectiveness of the suppression slices approach, which yielded only ~50% peak height reduction, ^31^P FID spectroscopy can be used to detect a drastic PCr/ATP ratio drop after infarction.

## Figures and Tables

**Fig. (2a,b):**
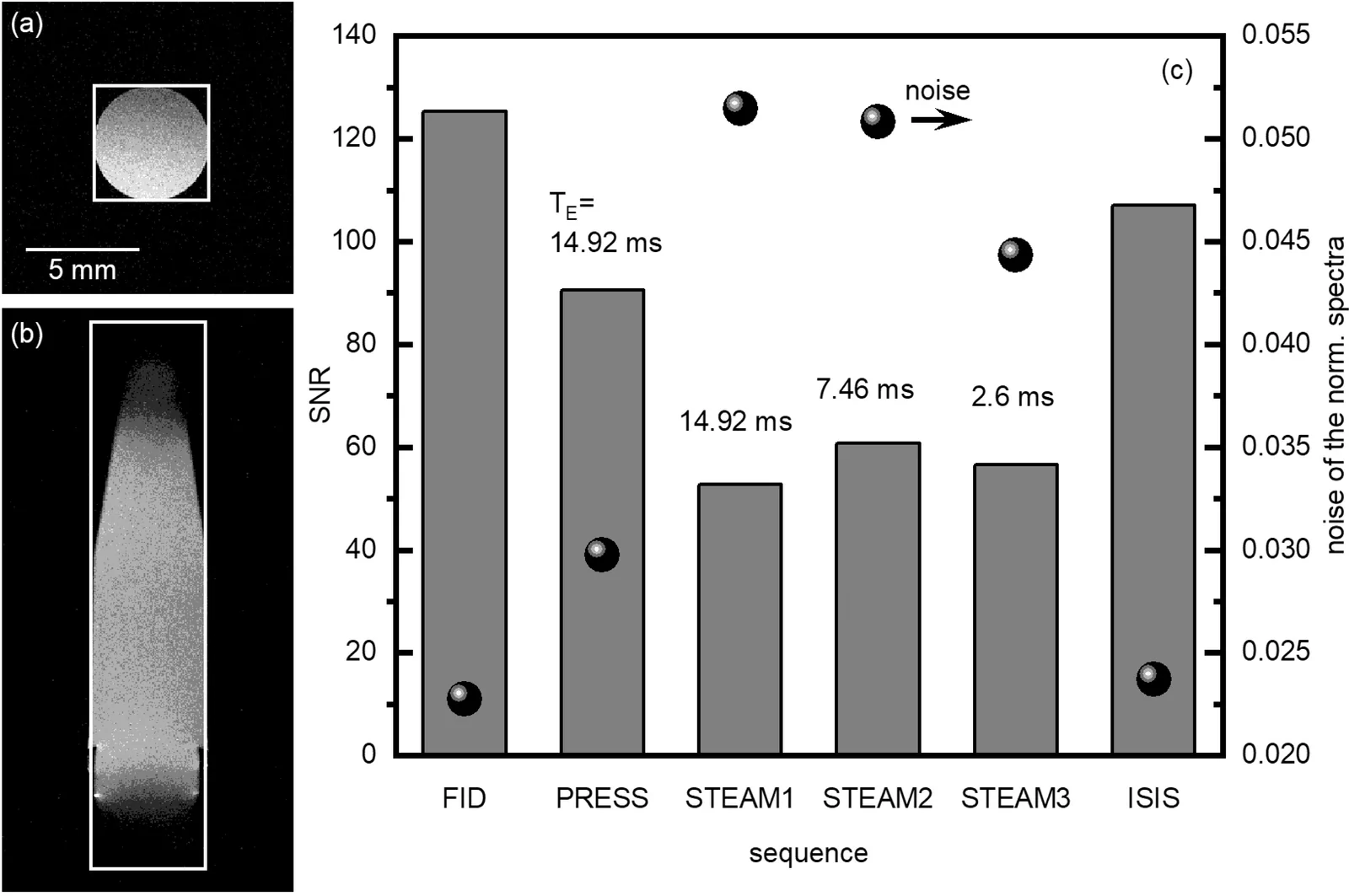
T2 reference images showing the phantom vial (0.2ml, 200mM PCr) and the voxel for localized spectroscopy. (**c**): Noise and SNR assessment of ^31^P spectra. The The SNR of PRESS and STEAM was significantly lower than that of FID or ISIS.

**Fig. (3):**
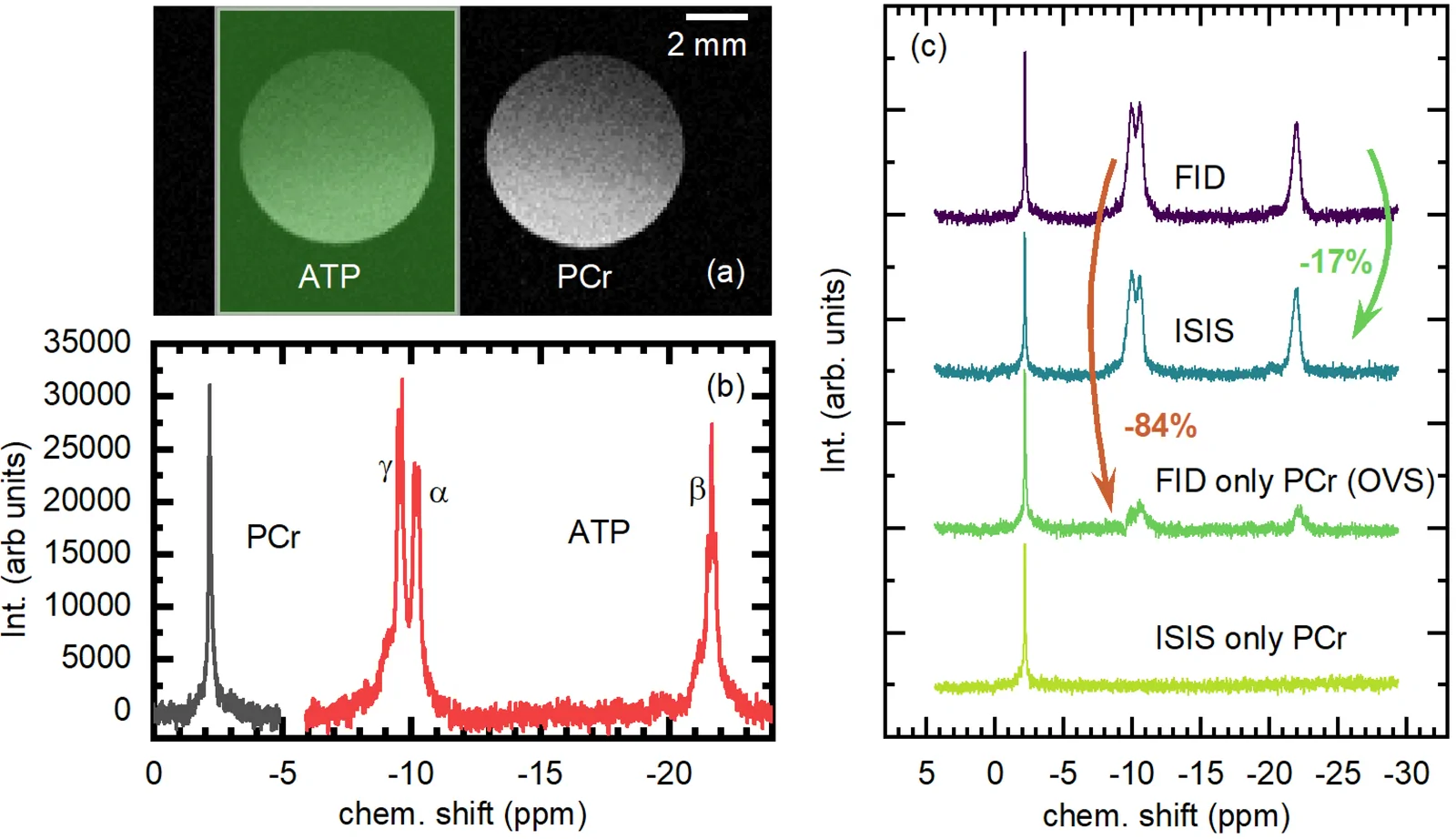
Comparison of selectivity between FID measurements with OVS and ISIS with different voxel locations. (**a**) Section view of the ATP and PCr phantoms. The suppression slice for the FID measurement is indicated as a green overlay. (**b**) Spectral signatures of the PCr and ATP solutions. Each of the two partial spectra was measured individually with a shim optimized for the corresponding sample. (**c**) FID and ISIS spectra of both samples with an optimized shim for the PCr sample. From top to bottom: the first and second spectra show FID and ISIS data recorded from the entire sample system; the third spectrum shows data of the PCr sample only, using OVS for the FID measurement; and the fourth spectrum shows the ISIS data with a voxel localized on PCr.

**Table 1:** Comparison of the parameters used for evaluating the sensitivity of different ^31^P spectroscopy sequences. FID and ISIS were also included for assessing selectivity. In FID and ISIS, T_E_ refers to the delay between the end of the excitation pulses and the start of data acquisition.

**Parameter**	**FID**	**STEAM**	**PRESS**	**ISIS**
T_acq time_ (s)	192	192	192	192
T_one acq_(s)	3	3	3	24
Averages	64	64	64	8
T_R_ (ms)	3000	3000	3000	3000
T_E_ (ms)	0.05	(1) 14.92 (2) 7.46 (3) 2.6	14.92 (with T_E_=T_E1_+T_E2_ and T_E1_=T_E2_)	0.05
Pulse shape (flip angle)	Block pulse (90°)	Calc pulse (90°/90°/90°)	Calc pulse (90°/180°/180°)	Block pulse (90°)/ calc pulse (180°)
Pulse duration (ms)	0.07	0.3	0.3 / 0.8	0.07 / 2.14

## Data Availability

The data that support the findings of this study are available from the corresponding author [S.P], upon reasonable request. Data are located in controlled access data storage at Rostock University Medical Centre.

## LIST OF ABBREVIATIONS

^31^P: Phosphorus
MRS: Magnetic resonance spectroscopy
ATP: Adenosine triphosphate
PCr: Phosphocreatine
MRI: Magnetic resonance imaging
FID: Free induction decay
NMR: Nuclear magnetic resonance
OVS: Outer volume suppression
SNR: Signal-to-noise ratio
STEAM: Stimulated echo acquisition mode
PRESS: Point-resolved spectroscopy
ISIS: Image-selected *in vivo* spectroscopy
CMRS: Chemical Shif MRS
CSI: Chemical Shift Imaging
TE: Echo time
VOI: Volume of Interest
TR: Repetition Time
GE: Gradient Echo
RF: Radiofrequency
LAD: Left Anterior Descending Coronary Artery
